# Investigating the prevalence of diabetic complications in overweight/obese patients: a study in a tertiary hospital in Malaysia

**DOI:** 10.1007/s13410-022-01131-x

**Published:** 2022-09-30

**Authors:** Shazwani Shaharuddin, Shobna Thuraisingam, Noorul Aimi Daud, Sarah Diyana Shafie, Sunanthiny Krishnan, Chia Siang Kow, Jamuna Rani Appalasamy, Dinesh Sangarran Ramachandram

**Affiliations:** 1 School of Pharmacy, Monash University Malaysia, Bandar Sunway, Selangor, Malaysia; 2 Pharmacy Department, Serdang Hospital, Selangor, Malaysia; 3 School of Pharmacy, International Medical University, Kuala Lumpur, Malaysia

**Keywords:** Prevalence, Diabetic complications, Overweight, Obese, Diabetes mellitus, Malaysia

## Abstract

**Background:**

In Malaysia, although diabetes accounts for more than 70% of all deaths, it is unclear how it relates to BMI and diabetic complications. This study aimed to investigate the prevalence of obesity and diabetic complications among diabetic patients in Malaysia.

**Materials and methods:**

A cross-sectional study using an existing clinical registry was performed from 1 January 2020 to 31 December 2020 at Hospital Serdang, Malaysia. Adult patients with type 2 diabetes mellitus had their medical records examined for disease complications, as reported by the patient at first contact with the DMTAC pharmacist.

**Results:**

The study comprised a total of 495 participants with an average HbA1c of 10.5%. About 91% (*n* = 451) of the 495 patients were obese/overweight. Around 37.8% (*n* = 187) of diabetic patients are between the ages of 50 and 59, and 59% (*n* = 292) have had diabetes for less than 10 years. A total of 8.5% (*n* = 42) and 9.7% (*n* = 48) consume alcohol and smoke, respectively. Around 29.9% (*n* = 148) had one other comorbidity (hypertension or dyslipidemia), and 63.4% (*n* = 314) had two comorbidities. Regarding the prevalence of complications, there were 18.9% (*n* = 94) who had myocardial infarction, 11.1% (*n* = 55) who had stroke, and 9% (*n* = 45) who had CKD. Age (adjusted OR = 1.03; 95% CI 1.00 to 1.07; *p* = 0.041) and hypertension (adjusted OR = 4.06; 95% CI 1.21 to 13.60; *p* = 0.023) were significantly related with the prevalence of complications in patients with diabetes.

**Conclusion:**

In our study, a BMI of more than 23 kg/m^2^ (obese/overweight) does not seem to be associated with the prevalence of complications. Age and hypertension, on the other hand, appear to be strong risk predictors of the incidence of complications. With the understanding of the recent outlook on diabetes, it is recommended that public education on the targeted population should be encouraged to negate these complications.

**Supplementary Information:**

The online version contains supplementary material available at 10.1007/s13410-022-01131-x.

## Introduction 

The number of diabetic patients worldwide has increased from 108 million in 1980 to 422 million in 2014 [1]. In 2019, diabetes was the ninth leading cause of death, with an estimated 1.5 million deaths directly caused by diabetes, and about half of all deaths occurred before the age of 70 years [1]. Obesity is one of the risk factors for the development of diabetes, and the number of individuals with obesity has tripled since 1975 [2]. An increase in body mass index (BMI) was associated with an increase in the risk of diabetes by about twofold in both men and women [3]. In Malaysia, although diabetes accounts for more than 70% of all deaths, it was unclear how it relates to BMI and diabetic complications [4]. This study aimed to investigate the prevalence of obesity and diabetic complications among diabetic patients in Malaysia.

## Methodology

### Data sources/study design

This cross-sectional study included data on all patients with type 2 diabetes registered with Serdang Hospital, Selangor, Malaysia. This study was conducted from January 2020 to December 2020. The patients with diabetes recruited in this study were more than 18 years of age and were seen in the specialized diabetic clinic between August 2008 and May 2020. Patients with incomplete data, less than 18 years old, pregnant, or having type I/gestational diabetes mellitus were excluded. An established clinical registry was used to examine the demographics of included patients. Baseline and follow-up clinical data were extracted by the investigator/coordinator, which included the patient’s gender, hemoglobin (Hb) A1c, age, BMI (as per Table 1), smoking history, alcohol consumption, and duration of diabetes. Comorbidities such as hypertension and dyslipidemia were recorded as well as diabetic complications (myocardial infarction [MI] and cerebrovascular accident [CVA], chronic kidney disease [CKD]). These complications were documented from clinical notes written on the patient’s first appointment at the diabetic clinic.

**Table 1:** BMI for the general population and Asian population [5]

	BMI for the general population	BMI for Asian
Underweight	< 18.5	< 18.5
Normal	18.5–24.9	18.5–22.9
Overweight	25–29.9	23–27.4
Obesity, class		
I	30–34.9	27.5–34.9
II	35–39.9	35–39.9
III	> 40	> 40

### Ethics

The study protocol was reviewed and approved by the National Medical Research Register (NMRR-19–3560-51,791).

### Sample size

Since no previous research was being conducted on the prevalence of complications among patients with diabetes in Malaysia, there were no references on the appropriate sample size for significant end results. A sample of 337 patients was deemed acceptable to elucidate significance in this study with a margin of error of 5%, based on a sample size calculation for a study of a fixed population and considering the annual number of inpatients at Serdang Hospital.

### Statistical analysis

The continuous variables were presented as mean ± standard deviation (SD) whereas categorical variables were presented as counts and percentages. Duration of diabetes was classified as less than 10 years and more than 10 years. Age categories were expressed as “ < 30,” “30–39,” “40–49,” “50–59,” and “60 and above.” To determine the association between independent variables and vascular complications, multiple logistic regression models were developed to estimate the adjusted odds ratios (ORs) and 95% confidence intervals (CIs), and independent variables with *p* ≤ 0.20 in simple logistic regressions were included. Statistical analysis was carried out using SPSS version 24.0 (SPSS Inc., Chicago, IL, USA).

## Results

Of 1227 diabetic patients, only 495 patients met the inclusion criteria and participated in this study. Table 2 depicts the demographic and clinical characteristics of the included patients by BMI categories. More than half (*n* = 282; 57%) of the patients were female. Mean HbA1c was 10.5%. Patients’ mean age was 52.2 (± 10.5) years, with patients aged between 50 and 59 years constituting the largest proportion (*n* = 187; 37.8%). The mean BMI was 31.0 kg/m^2^. Majority of the patients (*n* = 451; 91.1%) were either overweight/obese. Specifically, 129 (26.1%) patients were overweight and 322 (65.1%) patients were obese. Less than one-tenth of the patients were reported to consume alcohol (*n* = 42; 8.5%) and smoke actively (*n* = 48; 9.7%). The majority of the patients were diagnosed with diabetes for ≤ 10 years (*n* = 292; 59.0%) and had been living with two other comorbidities (*n* = 314; 63.4%).

**Table 2:** Demographics and clinical characteristics of the included patients (*n* = 495) by BMI categories

Patient characteristic	Total (*n* = 495)	Overweight/obese (*n* = 451)	Normal/underweight (*n* = 44)
	Frequency (%)	Frequency (%)	Frequency (%)
Gender			
Male	213 (43.0)	188 (41.7)	25 (56.8)
Female	282 (57.0)	263 (58.3)	19 (43.2)
Mean HbA1c	10.5	10.4	10.9
Mean age (years)	52.2	52.0	53.9
Age bracket (years)			
< 30	14 (2.8)	14 (3.1)	0 (0)
30–39	59 (11.9)	52 (11.5)	7 (15.9)
40–49	102 (20.6)	96 (21.3)	6 (13.6)
50–59	187 (37.8)	169 (37.5)	18 (40.9)
≥ 60	133 (26.9)	120 (26.6)	13 (29.5)
Mean BMI (kg/m^2^)	31.0	31.9	20.9
BMI (kg/m^2^)			
< 23	44 (8.9)	0 (0)	44 (100)
23–27.4 (overweight)	129 (26.1)	129 (28.6)	0 (0)
≥ 27.5 (obese)	322 (65.1)	322 (71.4)	0 (0)
Alcohol drinking	42 (8.5)	35 (7.8)	7 (15.9)
Current smoker	48 (9.7)	40 (8.9)	8 (18.2)
Duration of diabetes			
≤ 10 years	292 (59.0)	266 (59.0)	26 (59.1)
> 10 years	203 (41.0)	185 (41.0)	18 (40.9)
Hypertension (HPT)	388 (78.3)	361 (80.0)	27 (61.4)
Dyslipidemia (DLP)	388 (78.3)	355 (78.7)	33 (75.0)
Comorbidities			
0	33 (6.7)	28 (6.2)	5 (11.4)
1 (HPT/DLP)	148 (29.9)	130 (28.8)	18 (40.9)
2 (HPT + DLP)	314 (63.4)	293 (65.0)	21 (47.7)

**Fig. 1:**
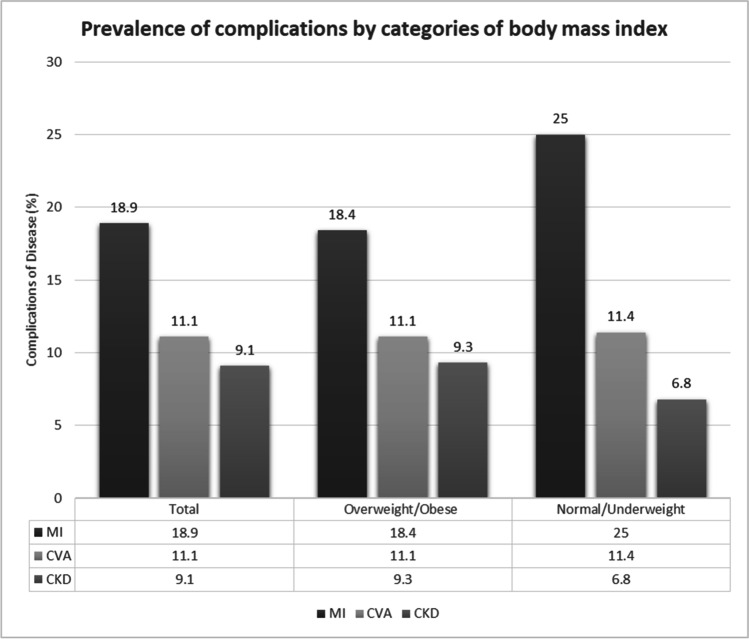
Prevalence of complications by categories of body mass index

**Fig. 2:**
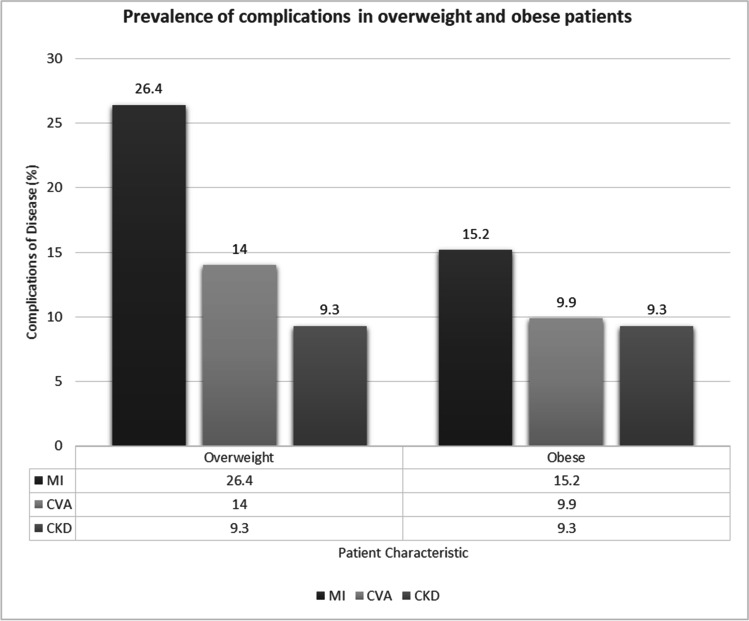
Prevalence of complications among overweight and obese patients

The prevalence of diabetic complications among the population in this study is manifested in Fig. 1 and Fig. 2. From the 495 patients included, 94 (18.9%) had myocardial infarction (MI), 55 (11.1%) had cerebrovascular accident (CVA), and 45 (9.1%) had chronic kidney disease (CKD). The prevalence of MI in the subgroup of patients with normal/underweight (*n* = 11/44; 25.0%) was higher than in patients with overweight/obesity (*n* = 83/451; 18.4%). Similarly, the prevalence of CVA was also noted to be higher in the subgroup of patients with normal/underweight (*n* = 5/44; 11.4%) compared to those in the overweight/obesity category (*n* = 50/451; 11.1%) although the difference was marginal. On the other hand, CKD was reported to be higher in the overweight/obesity subgroup (*n* = 42/451; 9.3%) compared to those within the normal/underweight (*n* = 3/44; 6.8%) category.

On the other hand, looking at the subgroup of patients with overweight/obesity, the prevalence of MI (*n* = 34/129; 26.4% versus *n* = 49/322; 15.2%) and CVA (*n* = 18/129; 14.0% versus *n* = 32/322; 9.9%) was higher in patients with overweight than patients were obese, but the prevalence of CKD (*n* = 12/129; 9.3% versus *n* = 30/322; 9.3%) was similar between patients with overweight and patients with obesity (Fig. 2).

Table 3 reports the multiple logistic regression analyses of factors associated with myocardial infarction, cerebrovascular accident, chronic kidney disease, and any of the three complications, respectively — independent variables with *p* ≤ 0.20 in simple logistic regressions (Table S1) were included. Male patients had a considerably higher risk of myocardial infarction than female patients (adjusted OR = 2.24; 95% CI 1.35–3.71; *p* = 0.002), and significantly increased with age (adjusted OR = 1.03; 95% CI 1.01–1.06; *p* = 0.020), and duration, patients with type 2 diabetes for > 10 years compared to patients with type 2 diabetes for ≤ 10 years (adjusted OR = 1.98; 95% CI 1.19–3.30; *p* = 0.009).

**Table 3:** Adjusted association between each of the micro- and macrovascular complications and potential risk factors

	MI		CVA		CKD		Any complication	
	Adjusted OR (95% CI)	*p* value	Adjusted OR (95% CI)	*p* value	Adjusted OR (95% CI)	*p* value	Adjusted OR (95% CI)	*p* value
Gender		0.002		0.371		-		0.316
Male	2.24 (1.35–3.71)		1.30 (0.73–2.31)		-		1.34 (0.76–2.37)	
Female	1		1		-		1	
HbA1c	-	-	0.85 (0.72–1.01)	0.064	-	-	-	-
Age	1.03 (1.01–1.06)	0.020	1.03 (0.99–1.06)	0.059	1.03 (1.00–1.07)	0.035	1.03 (1.00–1.07)	0.041
Alcohol drinking		0.099		-		-		-
Yes	1		-		-		-	
No	1.91 (0.89–4.12)		-		-		-	
Duration of diabetes		0.009		0.892		0.899		0.933
≤ 10 years	1		1		1		1	
> 10 years	1.98 (1.19–3.30)		1.04 (0.57–1.90)		1.04 (0.57–1.90)		1.03 (0.57–1.87)	
Hypertension		0.091		0.024		0.021		0.023
Yes	1.96		4.03		4.14		4.06	
No	1 (0.90–4.28)		1 (1.21–13.5)		1 (1.24–13.9)		1 (1.21–13.6)	
MI		-		-		0.791		-
Yes	-		-		0.91		-	
No	-		-		1 (0.45–1.84)		-	
CKD		0.050		-		-		-
Yes	2.02		-		-		-	
No	1 (1.00–4.08)		-		-		-	

The only factor significantly associated with the development of cerebrovascular accident in our cohort of patients with type 2 diabetes was the presence of hypertension (adjusted OR = 4.03; 95% CI 1.21–13.49; *p* = 0.024). On the other hand, the odds of chronic kidney disease significantly increased with age (adjusted OR = 1.03; 95% CI 1.00–1.07; *p* = 0.035) and is significantly higher in patients with concurrent hypertension (adjusted OR = 4.14; 95% CI 1.24–13.86; *p* = 0.021). Likewise, the odds of having any one of the three complications increased with age (adjusted OR = 1.03; 95% CI 1.00–1.07; *p* = 0.041) as well as in patients with concurrent hypertension (adjusted OR = 4.06; 95% CI 1.21–13.60; *p* = 0.023).

## Discussion

To the best of the authors’ knowledge, this is the first reported study within the literature that investigated the prevalence of obesity and the prevalence of complications among diabetic patients in Malaysia. The prevalence of combining both overweight and obesity in our study population with diabetes is 91%, which is relatively high as compared to what is being reported internationally [6–9]. Among the major complications of diabetes, myocardial infarction (MI) is the most common in our study cohort, a notable deviation from other countries, such as Riyadh, Sri Lanka, and Pakistan whereby chronic kidney disease is reported to be the most prevalent [6–9]. When patients were analyzed based on their BMI category (normal weight, overweight, and obese), the prevalence of MI remained the highest in all the categories, followed by CVA and CKD. However, the proportion of patients who suffered from MI was discovered to be greater in the subgroup of patients with normal/underweight in comparison with those in the overweight/obese category but it is not statistically significant. Contrary to the universally established premise [10, 11], overweight or obesity was not associated with an increased risk of diabetic complications in our study population. This atypical finding could possibly be attributed to the small sample size and the uneven distribution between the BMI categories in our study cohort. Larger and more comparable sample sizes between different BMI categories are therefore warranted to elucidate the association between weight and diabetic complications among the Malaysian population. A BMI of 23 kg/m^2^ was selected as the cut-off point for this study due to the higher proportion of type 2 diabetes and cardiovascular disease within the lower BMI group among Asians as compared to Caucasian (5). The prominence of cardiovascular complications in our patient population could have been influenced by the study location whereby Serdang Hospital is a reference center for cardiology and cardiothoracic surgery in Malaysia.

In this study, it was noted that male patients had a higher odd of getting MI compared to female patients, a finding that is consistent with the Framingham population whereby men were reported to have a twofold higher incidence of coronary heart disease and related mortality compared to women, although this gender gap attenuated with age [12]. One possible explanation for this gender disparity in the incidence of MI is the role of sex hormones in the pathogenesis of atherosclerosis. High levels of estradiol and progesterone during the premenopausal phase confer a cardioprotective effect on women by way of a more favorable lipid profile (i.e., higher high-density lipoprotein levels, lower low-density lipoprotein, and triglyceride levels) compared to men [13]. This effect however diminishes as women age into the postmenopausal phase, hence the similar rate of incidents in later stages of their lives.

Diabetes patients who had long-standing diabetes (i.e., more than 10 years) had higher odds of developing vascular complications. This is in line with a study that reported the prevalence of CVD increases from 6% for the duration between 0 and 10 years of diabetes to 10% and 30% in the duration of 10–20 years and more than 20 years, respectively [10]. Therefore, it is important that intervention is continuously done to ensure that the blood glucose is within the targeted range [14].

The risk of diabetic complications in our study population increases with age. Age has long been an established independent unmodifiable risk factor for myocardial infarction, stroke, and CKD [15–18]. On a broader perspective, this imposes a significant health and economic burden as Malaysia’s demographics steadily approach an aging population. As of 2021, 7.4% of the population in Malaysia is above the age of 65 [19]. Targeted efforts in mitigating risk factors that are modifiable such as smoking, consuming alcohol, weight, and physical activity would be particularly beneficial for this group of patients. Contemporaneously, efforts should also be directed at adopting the United Nations Decade of Healthy Ageing (2021–2030) program, a global collaboration aimed at fostering healthy aging [20].

The presence of comorbidities among patients with diabetes is very common and in our study population, about 30% had at least one comorbidity of either hypertension or dyslipidemia. About 63% reported having both of these comorbidities. As expected, patients with hypertension showed significantly increased odds of having diabetic complications. Alarmingly, hypertension was associated with a 3- to sixfold increase in the odds of developing CKD [21, 22]. This highlights the need for increased awareness, education, and optimal management in this group of patients.

With MI being the leading cause of death in Malaysia, diabetes mellitus together with other risk factors can add on to the increasing incidence of coronary heart disease [23]. It is made well known that the management of modifiable risk factors will help in dampening the progression of diabetic complications. This can be done through having optimal control of glucose, blood pressure, and cholesterol level. Management would include having a good dietary intake, being physically active, reducing consumption of alcohol, and abstinence from smoking. However, none of these will be able to be implemented without the support of a strong multidisciplinary team. Patient’s education also plays a vital role in increasing awareness on diabetic complications. A lot has been done in exploring the use of digital health in educating patients. Substantial evidence demonstrates the benefit of educating and continuously monitoring patients using digital platforms. The utilization of telecommunication technology has significantly developed in the past years and substantially penetrated the population with a wider usage. In the context of diabetic management, full blood glucose storing in the cloud, wearable technology to detect hypoglycemia, and telehealth/telemedicine are some of the technologies that have been implemented to assist diabetic patients [24, 25]. The issue that needs to be addressed here, however, would be the affordability of these technologies among the diabetic population and the effectiveness in reducing or perhaps delaying the incidences of complications among diabetic patients.

## Limitation

There are several limitations to this study that need to be considered. Due to physical restrictions imposed during the COVID-19 pandemic, data collection was restricted to only one tertiary hospital, thus limiting the generalizability of the results. Nevertheless, our data can be used as a starting point to develop a risk prediction tool for diabetes complications among the Malaysian population by running a future multicenter study that could further be implemented as a national strategy. This would bring a hope that the future study would be able to have equal distribution among obese and non-obese participants.

## Conclusion

Through this study, we found that there is no significant difference in the prevalence of complications between BMI categories. However, age and hypertension seem to be strong risk predictors of the incidence of complications in this cohort. Future studies could focus on age-specific associations between modifiable risk factors and other comorbidities, which may perhaps be a better predictor of its related outcomes.

## Supplementary Information

Below is the link to the electronic supplementary material.

Supplementary file1 (DOCX 15 KB)
