# Supplementary material for: Investigating the prevalence of diabetic complications in overweight/obese patients: a study in a tertiary hospital in Malaysia
Source: Int J Diabetes Dev Ctries. 2022 Sep 30:1–7. Online ahead of print. doi: 10.1007/s13410-022-01131-x (PMC9523188; doi:10.1007/s13410-022-01131-x)
Supplement: Supplementary file 1 — Supplementary file1 (DOCX 15 KB) [file 13410_2022_1131_MOESM1_ESM.docx]

**Table S1:** Simple logistic regression analyses

|  | **MI** | | **CVA** | | **CKD** | | **Any complication** | |
| --- | --- | --- | --- | --- | --- | --- | --- | --- |
|  | **Unadjusted OR (95% CI)** | **p value** | **Unadjusted OR (95% CI)** | **p value** | **Unadjusted OR (95% CI)** | **p value** | **Unadjusted OR (95% CI)** | **p value** |
| **Gender** |  | **<0.001** |  | 0.212 |  | 0.253 |  | **<0.001** |
| Male | 2.56 (1.61-4.06) |  | 1.43 (0.82-2.51) |  | 1.43 (0.77-2.64) |  | 2.05 (1.40-3.01) |  |
| Female | 1 |  | 1 |  | 1 |  | 1 |  |
| **HbA1c** | 1.03 (0.92-1.16) | 0.584 | 0.83 (0.70-0.98) | **0.025** | 1.06 (0.91-1.25) | 0.464 | 0.97 (0.88-1.08) | 0.588 |
| **Age** | 1.06 (1.03-1.08) | **<0.001** | 1.05 (1.02-1.08) | **0.003** | 1.12 (1.07-1.16) | **<0.001** | 1.08 (1.05-1.10) | **<0.001** |
| **BMI** |  | 0.289 |  | 0.956 |  | 0.584 |  | 0.549 |
| Normal/Underweight | 1 |  | 1 |  | 1 |  | 1 |  |
| Overweight/Obese | 0.68 (0.33-1.39) |  | 0.97 (0.37-2.58) |  | 1.40 (0.42-4.73) |  | 0.82 (0.43-1.57) |  |
| **Alcohol drinking** |  | **0.005** |  | 0.733 |  | 0.318 |  | 0.404 |
| No | 1 |  | 1 |  | 1 |  | 1 |  |
| Yes | 2.63 (1.34-5.17) |  | 0.83 (0.28-2.42) |  | 0.48 (0.11-2.04) |  | 1.32 (0.69-2.54) |  |
| **Current smoker** |  | 0.964 |  | 0.268 |  | 0.848 |  | 0.867 |
| No | 1 |  | 1 |  | 1 |  | 1 |  |
| Yes | 0.98 (0.46-2.11) |  | 0.51 (0.15-1.69) |  | 0.90 (0.31-2.63) |  | 0.95 (0.50-1.80) |  |
| **Duration of diabetes** |  | **<0.001** |  | 0.198 |  | **<0.001** |  | **<0.001** |
| ≤10 years | 1 |  | 1 |  | 1 |  | 1 |  |
| >10 years | 2.84 (1.79-4.52) |  | 1.45 (0.82-2.54) |  | 2.88 (1.52-5.45) |  | 2.34 (1.59-3.44) |  |
| **Hypertension** |  | **0.003** |  | **0.005** |  | **0.010** |  | **<0.001** |
| No | 1 |  | 1 |  | 1 |  | 1 |  |
| Yes | 3.05 (1.48-6.30) |  | 5.37 (1.64-17.54) |  | 13.56 (1.85-99.59) |  | 4.41 (2.38-8.16) |  |
| **Dyslipidaemia** |  | 0.850 |  | 0.513 |  | 0.917 |  | 0.923 |
| No | 1 |  | 1 |  | 1 |  | 1 |  |
| Yes | 0.95 (0.55-1.63) |  | 1.27 (0.62-2.62) |  | 0.96 (0.46-2.01) |  | 0.98 (0.62-1.54) |  |
| **MI** |  | - |  | 0.571 |  | **<0.001** |  | - |
| No | - |  | 1 |  | 1 |  | - |  |
| Yes | - |  | 1.22 (0.62-2.41) |  | 3.28 (1.72-6.26) |  | - |  |
| **CVA** |  | 0.571 |  | - |  | 0.323 |  | - |
| No | 1 |  | - |  | 1 |  | - |  |
| Yes | 1.22 (0.62-2.41) |  | - |  | 1.54 (0.65-3.65) |  | - |  |
| **CKD** |  | **<0.001** |  | 0.323 |  | - |  | - |
| No | 1 |  | 1 |  | - |  | - |  |
| Yes | 3.28 (1.72-6.26) |  | 1.54 (0.65-3.65) |  | - |  | - |  |
